# Chinese herbal extracts of *Rubia cordifolia *and *Dianthus superbus *suppress IgE production and prevent peanut-induced anaphylaxis

**DOI:** 10.1186/1749-8546-6-35

**Published:** 2011-09-30

**Authors:** Iván López-Expósito, Alexandra Castillo, Nan Yang, Banghao Liang, Xiu-Min Li

**Affiliations:** 1 Department of Pediatrics, Mount Sinai School of Medicine, New York, NY 10029-6574, USA; 2 Current Address: Institute of Food Science Research (CSIC-UAM), C/Nicolás Cabrera 9, 28049, Madrid, Spain

## Abstract

**Background:**

Peanut allergy is characterized by increased levels of peanut-specific IgE in the serum of most patients. Thus, the most logical therapy would be to inhibit the IgE production by committed B-cells. This study aims to investigate the unreported anti-IgE effects of Chinese herbal extracts of *Rubia cordifolia *(*Qiancao*) and *Dianthus superbus *(*Qumai*).

**Methods:**

Seventy herbal extracts were tested for their ability to reduce IgE secretion by a human B-cell line. Those with the lowest inhibitory concentration 50 (IC_50_) values were tested in a mouse model of peanut-anaphylaxis. Anaphylactic scores, body temperature, plasma histamine and peanut-specific-immunoglobulins were determined.

**Results:**

*Rubia cordifolia *and *Dianthus superbus *inhibited the *in vitro *IgE production by a human B-cell line in a dose-dependent manner and the *in vivo IgE *production in a murine model of peanut allergy without affecting peanut-specific-IgG1 levels. After challenge, all mice in the sham groups developed anaphylactic reactions and increased plasma histamine levels. The extract-treated mice demonstrated significantly reduced peanut-triggered anaphylactic reactions and plasma histamine levels.

**Conclusion:**

The extracts of *Rubia cordifolia *and *Dianthus superbus *inhibited the IgE production *in vivo *and *in vitro *as well as reduced anaphylactic reactions in peanut-allergic mice, suggesting potentials for allergy treatments.

## Background

Peanut allergy (PNA) is a worldwide health concern, particularly in developed countries. PNA accounts for approximately 80% of fatal and near-fatal food allergic reactions [1]. The prevalence of childhood PNA in the United States (USA) is currently at 1.4%, up from 0.8% in 2002 and 0.4% in 1997 [1]. Apart from strict avoidance of the peanut-containing foods, no satisfactory therapy is available to prevent or reverse this condition. Standard subcutaneous immunotherapy has been abandoned due to undesirable adverse reactions and marginal efficacy [2]. While peanut oral immunotherapy (OIT) is a promising therapeutic intervention for PNA [3], many questions remain, such as the risks of OIT compared with avoidance, dosing regimen issues, patient selection and post desensitization strategy [4]. Sublingual immunotherapy (SLIT) is a new method of treating food allergy, with few systemic reactions; however, only one study [5] determined the effect of SLIT on PNA. For these reasons, a safe and effective therapy for peanut allergy is urgently needed.

Research suggests that certain Chinese medicinal herbs may have the potential for treating allergy and asthma [6]. For the first time, our group developed a food allergy herbal formula (FAHF2) that blocks peanut-induced anaphylaxis in a mouse model [7,8]. A recent clinical trial demonstrated that the FAHF2 is safe and well-tolerated, with beneficial immunomodulatory effects *in vitro *[9].

Similar to other allergies, PNA is characterized by increased levels of peanut-specific IgE in the serum of most patients. Cross-linking of mast cell/basophil membrane cell-bound IgE antibodies by allergen results in the release of inflammatory mediators responsible for the signs and symptoms of PNA [10]. Omalizumab (Xolair) is the only available anti-IgE therapy which is one of the most logical therapies for PNA. Omalizumab effectively neutralizes IgE and may even cause apoptosis of committed B-cells by cross linking membrane IgE. However, relapse is likely if the antibody treatment stops [11,12]. While investigation of anti-allergic therapies from natural products sources has been increasing in the past years, the number of studies on reducing IgE production are limited [13].

The present study aims to investigate Chinese medicinal herbs that have previously unreported anti-IgE effects. Seventy herbal extracts were tested for their ability to reduce the IgE secretion by a human myeloma B-cell line. Those with the lowest IC_50 _values were then tested in a mouse model of peanut-anaphylaxis.

## Methods

### Herbs

All medicinal herbs used in this study were purchased from EFong Herbs Inc. (USA). These products were made by Gangdong Yifang Pharmaceutical Company Ltd. (China) and commercially available in the US *via *EFong Herbs Inc. All herbs were extracted with water and then concentrated and dried. The manufacturing processes and the product quality analyses are in accordance with GMP standards [14]. Each powdered extract was packaged and stored at room temperature under dark and dry conditions.

### High performance liquid chromatography (HPLC) fingerprints from Qiancao and Qumai

**Figure 1:**
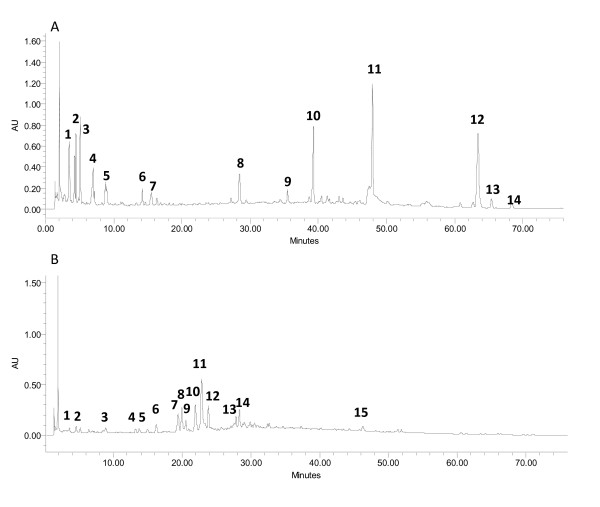
**HPLC chromatograms of *Qiancao *(*Rubia cordifolia*) and *Qumai *(*Dianthus superbus*)**. Panel A: *Qiancao*; Panel B: *Qumai*. HPLC conditions: column, Agilent Zorbax SB-C_18 _column (150 × 4.6 mm i.d.); flow rate, 1 mL/min; wavelength, 254 nm; mobile phase A, 0.1% formic acid, mobile phase B, acetonitrile. Data was analyzed using Waters Empower software.

HPLC fingerprints are recommended by the United States Food and Drug Administration as a means of standardization for botanical products. HPLC was carried out as previously described [9,15,16]. Briefly, 200 mg of *Qiancao *(QC) and *Qumai *(QM) extracts were dissolved into 2 mL mobile phase mixture consisting of 0.10% formic acid and acetonitrile (1:1). Each sample solution was filtered through a 0.2 μm filter (Whatman Inc., USA). Each sample (10 mL) was analyzed on a Waters Alliance 2695 HPLC system (Waters Corporation, USA) with a photodiode array detector (2996) (Waters Corporation, USA). The separation conditions were as follows: Zorbax SB-C_18 _column (150 × 4.6 mm; 5 μm particle size) from Agilent Technologies (USA); mobile phases: 0.10% formic acid (A) and acetonitrile (B); flow rate: 1.0 mL/min; detection wavelength: 254 nm. Linear separation gradient was from 2% of B to 48% for 75 minutes. Chromatographic results were acquired and processed with the Waters' Empower software (Waters Corporation, USA). All chemicals and solvents used were of HPLC grade (Fisher Scientific, USA). HPLC fingerprints of QC and QM are shown in Figure 1.

### U266 human B cell cultures and IgE measurement

Human U266B1 multiple myeloma cells (ATCC TIB-196™, American Type Culture Collection, USA) were cultured at 37°C in 5% CO_2_. RPMI 1640 medium, supplemented with 10% of fetal bovine serum (FBS), 1 mM sodium pyruvate, 1 × 10^-5 ^M 2-ME and 0.5% penicillin-streptomycin, was used as a culture medium. Cells were grown at an initial concentration of 2 × 10^5 ^cells/mL. Initially, all herbal extracts (Table 1) were added at Day 0 at 500 μg/mL and 100 μg/mL. At Day 6 the supernatants were harvested for total IgE assay. For those herbs with an IgE inhibition rate higher than 95% at both concentrations assayed, a dose-inhibition curve was performed.

Total IgE (T-IgE) was examined with a fluorescent enzyme immunoassay (ImmunoCAP FEIA, Phadia, Germany) and expressed in kU/L. The detection range of T-IgE was 2-2000 kU/L. Samples were measured undiluted, while samples with undetectable T-IgE were assigned an arbitrary value of 1 kU/L. The percentage of IgE inhibition was calculated as 100-[IgE concentration (sample treated) × 100/IgE concentration (sample untreated)]

### Cell viability assays for QC and QM cultures

The viability of control and treated cells was evaluated with the 3-(4, 5-dimethylthiazol-2-yl)-2, 5-diphenyltetrazolium bromide (MTT) assay in triplicates. Briefly, cells (2 × 10^4^) were incubated in 96-well microtiter plate containing 100 μL of the culture medium (RPMI 1640 medium supplemented with 10% FBS, 1 mM sodium pyruvate, 1 × 10^-5 ^M 2-ME and 0.5% penicillin-streptomycin) with or without tested compounds at 0, 3.125, 6.25, 12.5 25, 50, and 100 μg/ml). The MTT assay was performed after six days. Cells in each well were incubated at 37°C in 20 μg of the MTT (1 mg/mL) for four hours. After incubation, 150 μL of Dimethyl sulfoxide (DMSO) was added to each well. Absorbance of the mixture at 595 nm was determined with a microplate ELISA reader. The results were derived from three independent experiments.

### In vivo experimental protocol

**Figure 2:**
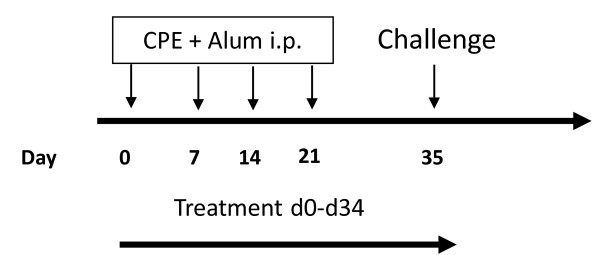
**Experimental protocol**. Mice were sensitized weekly for four weeks intraperitoneally (i.p.) with 200 μg of CPE and 2 mg of alum and then challenged i.p with 1000 μg CPE 2 weeks after the last sensitization. To determine whether QC and/or QM extracts prevent peanut anaphylactic reactions, we administered QC or QM at 2 mg or 4 mg intragastrically (i.g.) to a group of mice twice daily for five weeks beginning at Day 0 of the protocol (*n *= 5-8 mice per group).

Female C3He/J mice (6 weeks old) were purchased from Jackson Laboratory (USA). Standard guidelines for the care and use of animals were followed [17]. To generate a peanut allergy model, we sensitized the mice intraperitoneally (i.p.) each week with 200 μg of crude peanut extract (CPE) and 2 mg of alum in 0.5 mL of phosphate buffered saline (PBS) for four weeks, and then challenged (i.p.) them with 1000 μg CPE in 500 μL PBS two weeks after the last sensitization. To determine whether QC and/or QM prevent peanut anaphylactic reactions, we administered extracts of QC (2 mg) or QM (2 mg), or QC (4 mg) or QM (4 mg) in 0.5 mL of water intragastrically (i.g.) twice daily for five weeks beginning at Day 0 of the protocol. The dose was determined by a conversion table of equivalent human to animal dose ratios based on body surface area [18]. Additional peanut-sensitized mice received 0.5 mL water (i.g.) twice daily for five weeks as sham treatment controls (sham). Naïve mice served as normal controls (Figure 2).

### Assessment of systemic anaphylactic signs and measurement of core body temperatures

Anaphylactic signs were evaluated 30 to 40 minutes after the commencement of the challenge by two investigators using the following scoring: 0, no signs; 1, scratching and rubbing around the mouth and head; 2, puffiness and redness around the eyes and mouth, diarrhea, pilar erecti, reduced activity and/or decreased activity with increased respiratory rate; 3, wheezing, labored respiration and cyanosis around the mouth and tail; 4, no activity after prodding or tremor and convulsions and 5, death. Rectal temperatures were measured with a thermal probe (Harvard Apparatus, USA) every 15 minutes during the 30 minutes after the peanut challenge.

### Measurement of plasma histamine and mouse mast cell protease-1 (MMCP1) levels

Plasma was obtained 30 minutes after the challenge, histamine and MMCP1 levels were analyzed with an enzyme immunoassay kit as described by the manufacturers (Immunotech, France and Moredun Scientific, UK for histamine and MMCP1 measurements respectively).

### Measurement of serum antibodies

Retro-orbital venous blood was collected before the challenge. Sera were collected and stored at -80°C until analysis. Peanut-specific IgE and IgG1 levels were determined with a monoclonal antibody as previously described [19].

### Acute and sub chronic toxicity studies

The lethal dose 50 (LD_50_) protocol was designed as follows. Seven-week old mice were fed 12 times the highest therapeutic dose used in this experiment and observed for 12 and 24 hours; the LD_50 _was then calculated. Mice fed with water served as controls (sham). If no death occurred 12 and 24 hours after feeding, mice were observed for an additional 14 days. If no death occurred during this observation period, all mice were sacrificed. Samples were then collected for biochemical analyses, complete blood cell counts (CBC) and histological analyses. Biochemical analyses of blood urea nitrogen (BUN) as well as creatinine and alanine aminotransferase (ALT) were performed on a PROCHEM-V instrumentation (Synbiotics, USA) for the assessment of the kidney and liver functions respectively. For CBC testing, blood samples (20 μL) were collected and subjected to analysis by Multispecies Hematology Systems (CDC Technologies, USA). These assays were performed at Mount Sinai School of Medicine, Center for Laboratory Animal Sciences, where these assays are routinely performed to monitor the health of laboratory animals. Histological analysis of major organs (*ie *kidney, liver, heart, spleen, lung, stomach and intestine) was performed in a blinded manner.

### Statistical analysis

One-way or two-way ANOVA (analysis of variance) was performed followed by a Bonferroni correction for all pairwise comparisons if the data were approximately normal. If the data were not normally distributed, differences among multiple groups were analyzed by a Kruskal-Wallis ANOVA on ranks and Bonferroni correction was performed for all pairwise comparisons. *P *values of < 0.05, based on two-tailed tests, are considered statistically significant. Outliers were discarded based on Grubss test [20]. All statistical analyses were performed with GraphPad Prism (GraphPad Software Inc., USA).

## Results

### Anti-IgE screening for the Chinese medicinal herbs

**Figure 3:**
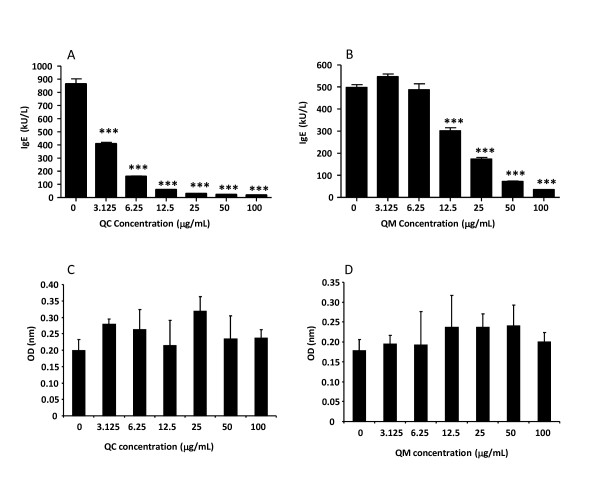
**Inhibitory effects of (a) QC and (b) QM on IgE production from U266 human B cells**. Cells were grown at an initial concentration of 2 × 10^5^cells/mL. QC and QM extracts were added at the indicated concentrations. At Day 6 the supernatants were harvested for total IgE assay. Cell viability after culturing U266 human cells with (c) QC or (d) QM was performed with MTT assay after six days of culture. Bars represent means ± SD of three independent experiments. ***P <0.001 *vs *untreated.

Seventy herbs extracts from our herbal repository with demonstrated anti-inflammatory actions were screened for potential anti-IgE properties *via *incubating them with an IgE producing human B-cell line (U266B1). Herbal extracts were added at Day 0 at concentrations of 500 μg/mL and 100 μg/mL. After six days of incubation, IgE levels in the supernatants were measured. Forty-nine of the 70 herbal extracts inhibited IgE production by less than 50% at 500 μg/mL. Nine inhibited between 50% and 80%, and 12 inhibited more than 80% (Table 1). At 100 μg/mL, the extracts of *Houpo *(*Magnolia officinalis*; 64%), *Huangbai *(*Phellodendron amurense*; 63.3%), *Huangqin *(*Scutellaria baicalensis*, 63.9%), QC (*Rubia cordifolia; *98.5%) and QM (*Dianthus superbus; *96.7%) inhibited more than 50%. Due to their remarkable inhibitory effects at 100 μg/ml, QC and QM were selected for further studies. First, dose response curves were determined as shown in Figure 3A and 3B. Both extracts, dose-dependently (3.125-100 μg/mL) inhibited IgE production, with IC_50 _values being 3.06 μg/mL (QC) and 12.33 μg/mL (QM). Furthermore, QC and QM did not affect the viability of U266B1 cells (Figure 3C and 3D), demonstrating that QC and QM have potent anti-IgE effect in a non-toxic manner.

**Table 1:** Selected Chinese medicinal plants with the percentage of IgE inhibition at the concentrations indicated

Pinyin name	Botanical name	Part used	% IgE inhibition 500 μg/mL	% IgE inhibition 100 μg/mL
Ai Ye	*Artemisiae argyi*	Leaves	69.5	14.4
Bai Guo Ren	*Ginkgo bilobae*	Seeds	0	0
Bai He	*Lilium brownii*	Bulb	0	0
Bai Hua She She Cao	*Heydyotis diffusa*	Whole	19.4	10.8
Bai Jiang Cao	*Patrinia scabiosaefolia*	Whole	17.2	0
Bai Shao	*Paeoniae lactiflora*	Root	25.2	5.7
Bai Tou Weng	*Pulsatillae chinensis*	Root	86.5	11.5
Bai Zhu	*Atractylodes Macrocephala*	Rhizome	10.3	0
Ban Bian Lian	*Lobelia chinensis*	Whole	21.0	3.9
Ban Xia	*Pinellia ternata*	Rhizome	15.6	11.5
Ban Zhi Lian	*Scutellaria Barbata*	Whole	39.1	16.6
Bei Sha Shen	*Adenophora tetraphylla*	Root	7.0	0
Bu Gu Zhi	*Psoraleae coryfolia*	Fruit	17.4	21.4
Cang Er Cao	*Xanthium sibiricum*	Whole	7.2	11.5
Cang Zhu	*Atractylodes lancea*	Root	19.9	8.6
Chai Hu	*Bupleurum chinense*	Root	31.3	11.1
Chan Tui	*Cryptotympana atrata*	Seeds	1.0	0
Che Qian Zi	*Plantago asiatica*	Seeds	14.4	12.9
Chuan Xin Lian	*Melia toosedan*	Root	67.5	18.6
Da Huang	*Rheum palmatum*	Root	71.29	5.2
Da Qing Ye	*Isatis tinctoria*	Leaves	37.34	12.88
Dan Shen	*Salvia miltiorrhiza*	Root	81.09	3.1
Dang Gui	*Angelica sinensis *	Root	10.8	0
Di Gu Pi	*Lycium chinense*	Bark	31.35	0
E Jiao	*Equus asinus*	Gelatin	9.9	0
Fu Ling	*Poria cocos*	Fruit body	11.16	10.3
Gan Cao	*Glycyrrhiza uralensis*	Root	7.2	11.5
Gan Jiang	*Zingiber officinalis*	Root	15.4	2
Gua Lou	*Trichosanthes kirilowii*	Whole	44.7	22.6
Hong Hua	*Carthamus tinctorius*	Flower	0	10.3
Hong Shen	*Panax ginseng*	Root	19.9	4.3
Hou Po	*Magnolia officinalis*	Bark	90.1	64.0
Huang Bai	*Phellodendron amurense*	Bark	96.6	63.3
Huang Qin	*Scutellaria baicalensis*	Root	94.4	63.9
Huang Yao Zi	*Dioscorea bulbifera*	Seeds	70.9	15.0
Ku Shen	*Sophora flavescens*	Root	1.0	0
Ling Zhi	*Ganoderma Lucidum*	Fruit body	14.7	9.6
Ma Bo	*Lasiosphera fenslii*	Fruit body	4.5	ND
Mai Dong	*Ophiopogon japonicus *	Root	4.3	4.3
Mu Dan Pi	*Paeonia suffruticosa*	Root bark	95.7	14.3
Mu Gua	*Chaenomeles lagenaria*	Fruit	10.8	1.6
Mu Li	*Ostrea gigas*	Shell	9.1	5.2
Qian Cao	*Rubia cordifolia*	Root	98.7	98.5
Qu Mai	*Dianthus superbus*	Whole	98.4	96.7
Rou Gui	*Cinnamomum cassia*	Bark	13.3	0
San Qi	*Panax notoginseng*	Root	0	0
Shan Ci Gu	*Cremastra variabilis*	Fruit body	6.2	5.4
Shan Dou Gen	*Sophora tonkineenis*	Root	15.1	0
Shan Zha	*Crataegus pinnatifida*	Fruit	0	0
Shan Zhu Yu	*Cornus officinalis*	Fruit	16.1	8.2
She Gan	*Belamcanda chinensis*	Rhizome	54.1	15.1
Sheng Jiang	*Drynaria fortunei*	Rhizome	90.6	14.2
Sheng Ma	*Cimicifuga foetida*	Rhizome/root	31.1	ND
Shi Chang Pu	*Acorus gramineus*	Rhizome	12.3	8.0
Si Gua Luo	*Luffa cylindrical*	Loofah	0	ND
Tian Dong	*Asparagus cochinchinensis*	Root	3.49	ND
Tian Hua Fen	*Trichosanthis kirilowii*	Root	12.78	ND
Tian Nan Xing	*Arisaema consaguineum*	Fruit	0	ND
Tou Weng	*Radix Pulsatibae*	Root	86.9	15.4
Tu Fu Ling	*Smilax glabra*	Rhizome	66.4	0
Wu Zhu Yu	*Evodia rutaecarpa*	Fruit	69.5	13.3
Xia Ku Cao	*Prunella vulgaris*	Flower	87.7	14.6
Xian He Cao	*Agrimonia pilosa*	Whole	71.7	0
Xiao Hui Xiang	*Foeniculum vulgare*	Whole	6.0	0
Yi Yi Renn	*Coix lachrymal jobi*	Seed	0	ND
Yu Mi Xu	*Zae mays*	Corn stigma	16.44	ND
Zhi Zi	*Gardenia jasminoides*	Seed	0	0
Zhu Ling	*Polysporus umbellatus*	Fruit body	8.14	ND
Zi Su Ye	*Perilla frutescens*	Flower	92.7	22.3

All Chinese herbal extracts listed were tested on the human B cell line (U266 B1) at 100 and 500 μg/ml. The pinyin and botanical names of the herbs tested in this study are based on Chinese Herbal Medicine: Materia Medica [32] and the Pharmacopoeia of the People's Republic of China [21].

### QC and QM suppressed peanut-specific IgE synthesis in an in vivo model of peanut-anaphylaxis

**Figure 4:**
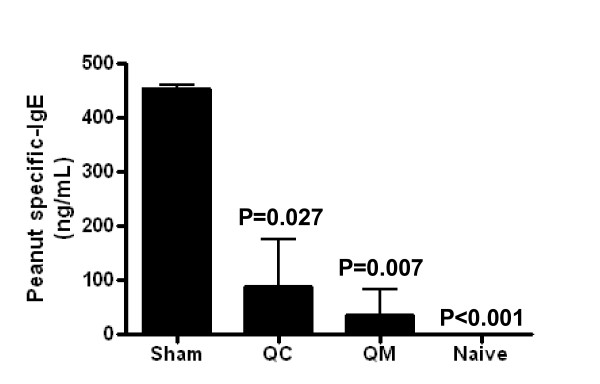
**Effect of QC and QM treatment on IgE production *in vivo***. Blood from each group of mice was collected one week before challenge. Peanut-specific IgE was measured by antigen-specific ELISA. Results are expressed as means ± SD of triplicates for each group (pooled samples; *n *= 5-8) P values are calculated *vs *sham.

Since sensitization of mast cells with IgE is an essential mechanism in the anaphylaxis cascade, we evaluated the effect of QC and QM on peanut-specific IgE production in an *in vivo *model of peanut-anaphylaxis (Figure 2) and found that QC (4 mg) and QM (4 mg)-treated mice showed reductions of 80.47% (*P = *0.027) and 92.34% (*P = *0.007) respectively in their peanut-specific IgE levels compared with sham-treated mice one week before the time of challenge (Figure 4). Peanut-specific-IgG1 levels were slightly reduced in the QC (4 mg) and QM (4 mg) treatment group, but the difference was not statistically significant at this time point (9.91 × 10^6^ng/mL ± 720345 for sham *vs *8.73 × 10^6^ng/mL ± 425234 for QC (4 mg) and sham *vs *7.83 × 10^6^ng/mL ± 200283 for QM (4 mg).

### QC and QM decreased peanut triggered anaphylactic reactions in a mouse model

**Figure 5:**
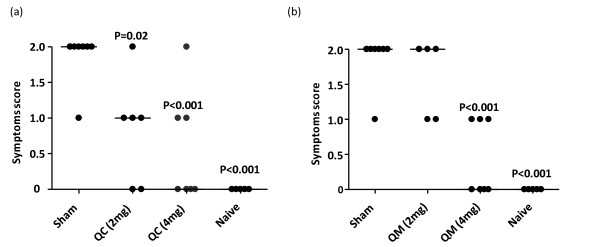
**Effect of (a) QC and (b) QM treatment on peanut-induced anaphylactic symptoms**. Anaphylactic scoring was done as described in the methods section. Symbols indicate individual mice from two sets of experiments (*n *= 5-8). Bars are medians of scores. P values are calculated *vs *sham.

In order to investigate whether QC and QM can prevent anaphylaxis *in vivo*, we used peanut as a model antigen to test the effects of QC and QM on peanut-induced anaphylactic reactions. A mouse model of peanut allergy was established (Figure 2). After challenge at Day 35, all sham-treated mice developed anaphylactic reactions (median score 2, Figure 5A and 5B). By contrast, mice treated daily with QC (4 mg) or QM (4 mg) exhibited significantly reduced anaphylactic symptoms (median score 0; *P <*0.001; Figure 5A and 5B). At a dose of 2 mg, only QC treated mice exhibited reduced anaphylactic reactions (median score 1; *P *= 0.020; Figure 5A).

### QC and QM prevented decreases in body temperature after peanut challenge

**Figure 6:**
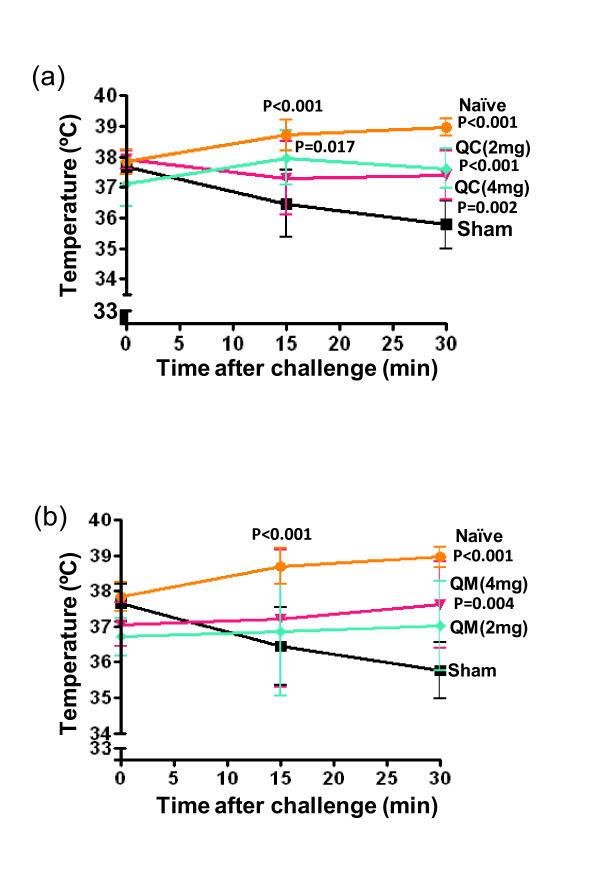
**Effect of (a) QC and (b) QM treatment on core body temperatures during challenge using a rectal thermometer**. Each data point indicates group means ± SD of individual mice from two sets of experiments (*n *= 5-8).P values are calculated *vs *sham.

Core body temperature drops during systemic anaphylaxis. We used rectal temperature measurement at 30 minutes after challenge as an objective measurement of anaphylaxis. As shown in Figure 6A and 6B, mean temperatures of the sham-treated mice were significantly lower than those of the naïve mice (35.77 ± 0.79°C *vs *38.96 ± 0.28°C; *P <*0.001). Similarly mean temperatures in the QC- and QM-treated mice were significantly higher than in the sham-treated mice, namely 37.39 ± 0.79°C for QC (4 mg) and 37.62 ± 1.22°C for QM (4 mg) (Figure 6A and 6B) (*P *= 0.0018 for QC and *P = *0.004 for QM). As the strongest activity was found at the dose of 4 mg/day/mouse, the rest of the experiments used this dose.

### QC and QM prevented histamine release after peanut challenge

**Figure 7:**
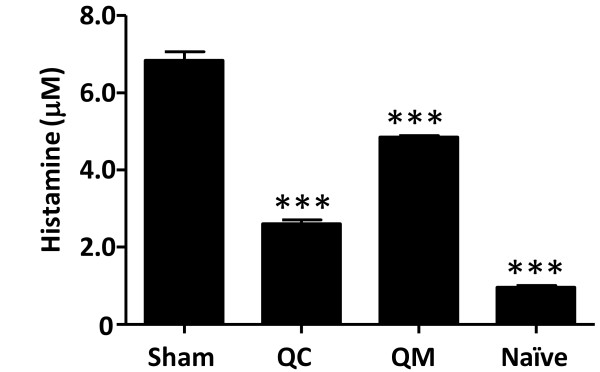
**Effect of QC and QM treatment on histamine release after challenge measured by ELISA**. Plasma from each group of mice was collected 30 minutes post-challenge. Results are expressed as means ± SD of triplicates for each group (pooled samples; *n *= 5-8). ***P <0.001 *vs *sham.

Anaphylactic scores in this model are associated with plasma histamine levels. We determined histamine levels 30 minutes after challenge. As shown in Figure 7A, plasma histamine levels were markedly elevated in the sham-treated mice compared with naïve mice (sham *vs *naïve, mean, μM: 6.843 ± 0.3970 *vs *0.954 ± 0.085; *P <*0.001). By contrast, histamine levels in the QC- and QM-treated mice were significantly lower than those in the sham-treated mice (*P <*0.001). Plasma MMCP1concentrations were also measured as an additional marker of mast cell degranulation. We found a significant decrease in MMCP1 levels in the mice treated with 4 mg of QM (sham *vs *QM, mean, ng/mL: 519.8 ± 212.3 *vs *238.6 ± 224.5; *P *= 0.042); however, no significant differences were found in the QC-treated mice.

### Safety of QC and QM

In a preliminary safety assessment, we performed LD_50 _testing. No mouse died within the 12 hours after they were administered the effective mouse daily dose of QC or QM (*n *= 10), nor did any of them die during the subsequent two weeks.

To further assess safety, we collected blood samples specimens from mice two weeks after they were fed and subjected to biochemical analysis of BUN and ALT for the assessment of liver and kidney functions respectively. All results were within the normal range (Table 2). Moreover, histological examination of the major organs did not reveal any abnormalities.

**Table 2:** Biochemical assays and CBC testing

	Biochemical assays		CBC testing			
	
	BUN (mg/dL)	ALT (U/L)	WBC (K/μL)	RBC M/μL	Hb (g/dL)	PLT (K/μL)
Qian Cao	23.2 ± 4.9	28.6 ± 6.9	3.3 ± 1.5	9.9 ± 1.4	14.7 ± 0.7	769.2 ± 222.9
Qu Mai	20.6 ± 1.8	26.4 ± 1.4	4.9 ± 1.7	8.6 ± 0.7	14.6 ± 0.4	698 ± 176.8
Sham	24.5 ± 5.3	31.6 ± 3.6	6.6 ± 1.9	9.2 ± 0.9	14.8 ± 0.6	700.4 ± 195.9

Reference	9 - 36	22 - 400	1.8 - 10.7	6.4 - 9.4	11.0 - 15.1	592 - 2972

Mice were euthanized with CO_2_, and blood was collected by cardiac puncture. Biochemical assays and CBC testing were conducted as described in Methods. Results are mean ± SD of 5-10 mice from each group.

## Discussion

After screening 70 herbs extracts with previously reported anti-inflammatory properties, we found that QC and QM extracts markedly inhibited IgE production by a B-cell human cell line over a concentration range of 100 μg/mL to 3.125 μg/mL. The inhibition was not due to toxicity because proliferation assays showed no effect, even at the highest concentrations used (Figure 3C and Figure 3D). QC root is listed in the Chinese Pharmacopoeia for the treatment of arthritis, chronic bronchitis, uterine hemorrhage and uteritis [21]. Recent studies have shown that QC roots have antibacterial, antioxidant and anti-inflammatory activities [22-24]. QM is an important Chinese medicinal herb used as a diuretic and an anti-inflammatory agent for the treatment of urinary infections, carbuncles and carcinomas [21]. To our knowledge, we are the first to report the anti-IgE properties of both herbs. Kim, Lee, Won, Park, Chae, Kim & Baek; Kim, Kim & Park and Kim & Moon reported the IgE inhibitory effect of some other herbs such as *Asiasari Radix*, *Poncirus trifloliata *and *Siegesbeckia glabrescens *using the same cell line as in our experiments; however, the concentrations required for the inhibitory effects were higher than those in our experiments [25-27]. Sugahara, Nishimoto, Morioka, Nakano & Nakano [28] identified anti-IgE activity of extracts of *Sorghum bicolor *(L.) Moench. In their experiments, the extracts suppressed IgE production by the human myeloma cell line U266 in a dose-dependent manner but did not affect the IgG production by mice splenocytes *in vitro*. We demonstrated a similar effect in our *in vivo *studies, in which mouse serum peanut-specific IgG1 levels did not significantly differ between the groups, suggesting that the effects of QM and QC are IgE specific.

PNA accounts for approximately 80% of the fatal and near-fatal anaphylactic reactions to foods [29]. As peanut-induced anaphylaxis is IgE-mediated, we tested the effects of QC and QM in a well established mouse model of PNA. Mice in these experiments exhibited less severe symptoms than in a previous study [8] perhaps because mice in these studies were i.p. sensitized with crude peanut extract whereas we used i.g. feeding of ground whole peanut and cholera toxin in our previous studies. Mice's sensitivity to antigen may also differ over time. Recent studies [30,31] showed that longer sensitization protocols were required to produce the same anaphylactic responses as in a previous study [8].

Both QC and QM prevented peanut-induced anaphylaxis. This protection could be a direct consequence of the reduced peanut IgE levels induced by the QC and QM treatment. Furthermore, significantly less histamine release was observed in the treated animals. The decrease may be attributed to reduced IgE production by B-cells, leading to decreased availability of IgE for participation in mast cell activation and consequently mast cell degranulation upon antigen challenge. In this model the severity of anaphylactic symptoms is correlated with mast cell histamine release. Both QM and QC significantly reduced plasma histamine levels following peanut challenge of PNA mice, thereby protecting against systemic anaphylaxis. Histamine release is a central mechanism involved in the IgE-mediated type I hypersensitivity reactions in humans and also an important parameter for evaluating the effects in this model. Moreover, QM but not QC also produced significant suppression of MMCP1 release although both QM and QC similarly suppressed systemic anaphylaxis, suggesting that MMCP1may not be the most appropriate marker of systemic anaphylaxis in this model.

## Conclusion

*Qiancao *(*Rubia cordifolia*) and *Qumai *(*Dianthus superbus*) extracts inhibit the IgE production by plasma cells *in vitro *and in mice in a non-toxic manner. This, at least in part, may be responsible for the observed protection against anaphylaxis. Further research is warranted to investigate the molecular mechanisms underlying the inhibitory effects and to identify the active compounds responsible for these effects. More importantly, controlled clinical studies are required to further ensure the safety and efficacy for the use of these herbal products for human food allergy.

## Abbreviations

ALT: alanine aminotransferase; BUN: blood urea nitrogen; CBC: complete blood cells counts; CPE: crude peanut extract; DMSO: dimethyl sulfoxide; FAHF2: food allergy herbal formula 2; FBS: fetal bovine serum; Hb: Hemoglobin; i.g.: intragastric; i.p.: intraperitoneal; IC50; inhibitory concentration 50; LD_50_: lethal dose 50; MMCP1: mouse mast-cell protease 1;MTT: 3-(4, 5-dimethylthiazol-2-yl)-2, 5-diphenyltetrazolium bromide; OIT: oral immunotherapy; PLT: platelets;PNA: peanut allergy; QC: *Qiancao*; QM: *Qumai*; RBC: red blood cells: SLIT: sublingual immunotherapy; WBC: white blood cells.

## Competing interests

The authors declare that they have no competing interests.

## Authors' contributions

XML conceived and designed the study and finalized the manuscript. ILE performed the experiments, data acquisition, result interpretation and manuscript preparation. AC and BHL performed the experiments. NY collected and authenticated the plant samples. All authors read and approved the final version of the manuscript.
